# Plasma Ceramide Concentrations in Full-Term Pregnancies Complicated with Gestational Diabetes Mellitus: A Case-Control Study

**DOI:** 10.3390/metabo12111123

**Published:** 2022-11-16

**Authors:** Maria Lantzanaki, Stavroula Veneti, Gesthimani Mintziori, Olga Begou, Panagiotis D. Pappas, Helen Gika, Dimitrios G. Goulis, Helen Bili, Eleftheria Taousani, Dimitrios Vavilis

**Affiliations:** 1 1st Department of Obstetrics and Gynecology, Medical School, Aristotle University of Thessaloniki, 54124 Thessaloniki, Greece; 2 Laboratory of Analytical Chemistry, Department of Chemistry, Aristotle University of Thessaloniki, 54124 Thessaloniki, Greece; 3 Biomic AUTh, Center for Interdisciplinary Research and Innovation (CIRI-AUTH), Balkan Center, 10th km Thessaloniki-Thermi Road, 57001 Thessaloniki, Greece; 4 Laboratory of Forensic Medicine & Toxicology, School of Medicine, Aristotle University of Thessaloniki, 54124 Thessaloniki, Greece; 5 Department of Midwifery, Faculty of Health Sciences, International Hellenic University, Alexander Campus, 57001 Thessaloniki, Greece; 6 Medical School, University of Cyprus, Nicosia 2408, Cyprus

**Keywords:** ceramides, gestational diabetes mellitus, prognostic factors, pregnancy

## Abstract

Ceramides, a sphingolipid group that acts as a messenger in cellular differentiation, proliferation, apoptosis and senescence, have been associated with cardiovascular disease and type 2 diabetes. The evidence for an association between ceramides and gestational diabetes mellitus (GDM) is scarce. This case-control study aimed to compare women with GDM with healthy, pregnant women in terms of plasma ceramide concentrations at the time of delivery. Ninety-two pregnant women were included in this case-control study, 29 in the GDM group and 63 in the control group. All women were admitted to a tertiary academic hospital for a full-term delivery. Liquid chromatography-tandem mass spectrometry (LC-MS/MS) was applied for the quantification of four molecular ceramides, namely Cer d18:1/16:0 (Cer16:0), Cer d18:1/18:0 (Cer18:0), Cer d18:1/24:0 (Cer24:0) and Cer d18:1/24:1 (Cer24:1) in plasma samples. The raw chromatographic data obtained from the LC-MS/MS analysis were processed using Analyst SCIEX (AB Sciex Pte. Ltd., USA). In a univariate statistical analysis, Cer24:0 concentration was significantly lower in the GDM group compared with the control group (*p* = 0.01). The present study demonstrated lower Cer24:0 concentrations in pregnancies complicated by GDM. Further prospective studies are required to enhance the results of this study.

## 1. Introduction

Gestational diabetes mellitus (GDM) complicates 7.8% (7.2–8.4%) of pregnancies in Europe and can affect the fetus, the mother, and the pregnancy outcome [1]. It is defined as glucose intolerance diagnosed for the first time during pregnancy. There is an increase in the GDM prevalence due to the increased rate of obesity in western countries and the older age of pregnant women [2,3]. Risk factors for GDM include race, age, body mass index (BMI), history of GDM, family history of diabetes mellitus and polycystic ovary syndrome (PCOS) [4]. The main GDM complications are hypertension, preeclampsia, fetal macrosomia, shoulder dystocia and fetal death. Newborns can suffer from hypoglycemia, respiratory distress syndrome and hyperbilirubinemia [5].

Ceramides, a sphingolipid group, act as messengers in cellular differentiation, proliferation, apoptosis and senescence [6]. They are located in the external layer of the plasma membrane and are messengers of the sphingomyelin transmembrane signaling pathway. Apoptosis and differentiation inducers, damaging agents and inflammatory cytokines can increase the production of ceramides, and thus the plasma concentrations of their free forms [7]. Plasma ceramides are biomarkers of cardiovascular disease, cancer, Alzheimer’s disease and type 2 diabetes mellitus (T2DM) [8,9,10]. Elevated plasma ceramide concentrations have been associated with preeclampsia and preterm delivery [11,12].

The evidence on an association between ceramides and GDM is scarce. Placental ceramide concentrations in women with GDM treated with insulin were higher compared with placentas of uncomplicated pregnancies or placentas of women with GDM treated with diet [13]. There are no publications concerning plasma ceramide concentrations in full-term pregnancies complicated by GDM.

Our study hypothesis is that plasma ceramides in pregnant women with GDM should be elevated, because of the similar mechanisms of GDM with T2DM.

The present study aimed to investigate if plasma ceramide concentrations of N-Palmitoyl-D-*erythro*-Sphingosine [Cer (d18:1/16:0)–Cer16:0], N-Stearoyl-D-*erythro*-Sphingosine [Cer (d18:1/18:0)–Cer18:0; N-lignoceroyl-D-*erythro*-sphingosine [Cer (d18:1/24:0)–Cer24:0] and N-Nervonoyl-D-*erythro*-Sphingosine [Cer (d18:1/24:1)–Cer24:1] differ between pregnancies complicated by GDM and uncomplicated pregnancies at delivery. We opted to study these four ceramides because they are the most studied in the literature, both in T2DM and in pregnancy complications. More specifically, Cer16 and Cer18, which are shorter-chain saturated fatty acid ceramides, were positively associated with T2DM in the Dallas Heart Study [9], whereas Cer24 and Cer 24:1 are two of the most abundant naturally occurring ceramides.

To the best of our knowledge, this is the first study that attempts to answer this research question

## 2. Materials and Methods

### 2.1. Study Characteristics

This is a case-control study conducted in the outpatient clinic of a tertiary academic hospital.

### 2.2. Patients

Ninety-two pregnant women participated in the study. All of these women were recruited between August 2020 and August 2021. The patients were enrolled consecutively. Of them, 29 were diagnosed with GDM during their pregnancy (GDM group), and 63 had uncomplicated pregnancies (control group). The latter were matched to the former for gestational age. All women were admitted to our department to deliver as term pregnancies (>37 gestational weeks). The diagnosis of GDM was set according to the International Association of the Diabetes and Pregnancy Study Groups (IADPSG) criteria [14]. Exclusion criteria for both groups were preterm delivery (<37 gestational weeks), type 1 (T1DM) or T2DM, and personal history of chronic diseases and obstetrical complications, such as preeclampsia, IUGR, hypertension or chorioamnionitis. All women were of Caucasian origin.

### 2.3. Methods

All patients provided written informed consent before entering the study. After their admission, a personal, obstetrical, and family history was taken. A blood sample was obtained and centrifuged for 15 min. The plasma used for measuring ceramides was removed and stored at −80 °C. For every woman, the following parameters were recorded: age, gestational age, parity, smoking, body mass index (BMI), gestational weight gain (GWG), oral glucose tolerance test (OGTT) values, delivery type, neonatal birth weight, Apgar score, blood pressure, creatinine, urea, aspartate aminotransferase (SGOT), alanine aminotransferase (SGPT), uric acid, platelets, and presence/absence of protein in urine samples. All parameters were measured at the academic hospital’s laboratories, using established techniques.

### 2.4. Sample Preparation

Plasma samples were left to thaw at room temperature before sample preparation. A volume of 100 μL of plasma sample was diluted with 20 μL of IS (mix of isotope-labeled ceramides). The sample was vortex mixed for 2 min, and 1 mL of CH_2_Cl_2_:MeOH, 2:1 *v*/*v* was added. The sample was vortex mixed for 5 min and centrifugated at 6700× *g* for 15 min at 4 °C. A volume of 900 μL of the lower organic phase was evaporated to dryness under vacuum, and the dry residue was reconstituted with 50 μL of IPA:MeOH, 1:1 *v*/*v*. The final extract was subjected to LC-MS/MS analysis.

### 2.5. Chemicals and Materials

N-palmitoyl-D-*erythro*-sphingosine (Cer16:0), N-stearoyl-D-*erythro*-sphingosine (Cer18:0), N-lignoceroyl-D-*erythro*-sphingosine (Cer24:0) and N-nervonoyl-D-*erythro*-sphingosine (Cer24:1) were obtained from Avanti Polar Lipids, Inc., Alabaster, AL, while their respective isotope-labeled standards used as an internal standard (IS) were purchased as a mixture (LIPIDOMIX^®^ Mass Spec Standard solution) from Avanti Polar Lipids, Inc., Alabaster, AL, USA.

### 2.6. LC-MS/MS Analysis

All samples were analyzed using a previously developed and validated Liquid Chromatography-tandem Mass Spectrometry (LC-MS/MS) method for the simultaneous determination and quantification of the four ceramides, as described by Begou et al. [15]. Briefly, chromatography was performed by an Alliance HT Waters 2790 (Waters Corporation, Milford, MA, USA) system, on a ReproShell ODS-3 (2 mm × 50 mm, 2.7 μm) (Dr. Maisch GmbH, Ammerbuch-Entringen, Germany) column under reversed phase conditions. Intra-day accuracy and precision of the method were between 80–111% and 0.05–10.2% for all ceramides, respectively, while inter-day accuracy and precision ranged from 87.8–106% to 2.2–14%, respectively. The lower limit of quantification (LOQ) was 2.3 ng/mL for Cer16:0 and Cer18:0 and 1.4 ng/mL for Cer24:0 and Cer24:1. To assess the analytical batch precision, a quality control (QC) sample was used, and prepared by mixing equal volumes of all the analyzed samples (pooled plasma sample). The QC sample was analyzed six times at the beginning of the analytical run and then every 10 real samples.

### 2.7. Statistical Analysis

Sample size estimation. No data exists on plasma ceramide concentrations at term in pregnancies complicated by GDM. As the most similar pathology to GDM reporting on ceramide concentrations is preeclampsia, a relevant study was used to define the sample size [11]. In patients with preeclampsia, the Cer24:0 concentration was 2.588 ± 0.574 nmol/mL (0.168 ± 0.037 mg/dL) (mean ± standard deviation) and 4.477 ± 1.986 nmol/mL (0.291 ± 0.129 mg/dL) in uncomplicated pregnancies. With a type 1 error of 5% and a type 2 error of 10%, a sample of 28 patients (14 GDM cases and 14 controls) was calculated as appropriate. Nevertheless, as this was a rough estimation of the expected differences between the groups, it was decided that larger sample sizes were to be recruited in both groups: 29 in the GDM group and 63 in the control group.

### 2.8. Statistical Methods

Quantitative results of ceramides were evaluated using the statistical software GraphPad Prism version 7.00 for Windows (GraphPad Software, La Jolla California USA, www.graphpad.com, accessed on 30 January 2022). Data were assessed for normal distribution based on D’Agostino–Pearson (95% de) statistical test. A two-tailed *t*-test (Mann–Whitney test) with unequal variance and a threshold of *p*-value < 0.05 was performed. Area under the receiver operating characteristic (AUC-ROC) curves and box plots were constructed for the four ceramides. The correlation of all analytes and patients’ personal history, BMI, GWG and other laboratory parameters was assessed using the Spearman correlation.

## 3. Results

A total of 92 women were included in the study, 29 in the GDM group and 63 in the control group. The demographic characteristics of the study participants are shown in Table 1. The mean maternal age in the GDM group was 33.0 ± 4.5 and in the control group 30.0 ± 6.6 years (*p* = 0.029). Cesarian section was more likely in women with GDM than in controls (58.6% vs. 47.6%, respectively, *p* = 0.327).

The percentage of relative standard deviation (%RSD) of all ceramides in the QC sample was <10%, verifying a satisfactory system precision.

Ceramide concentrations were quantified in all samples and mean values [± standard deviation (sd)] were calculated (Table 2).

**Figure 1:**
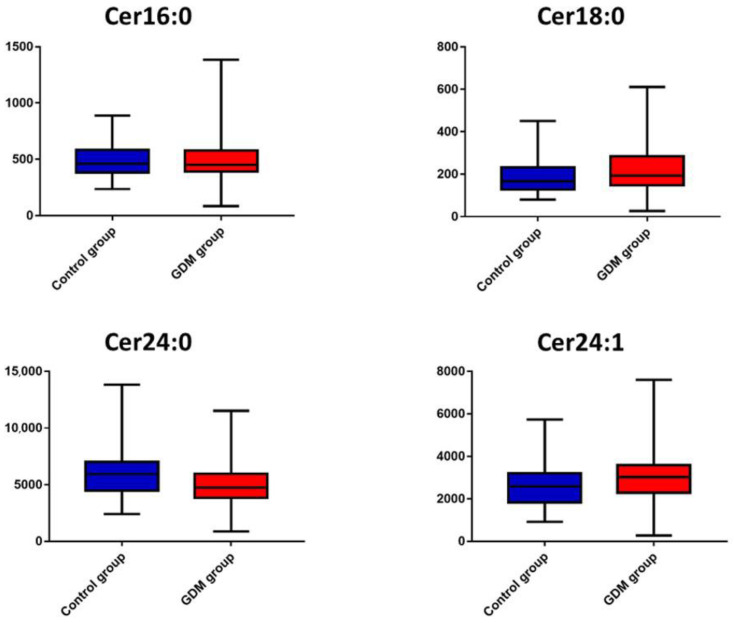
Box plots of the four measured ceramides in GDM (red) and control (blue) groups.

**Figure 2:**
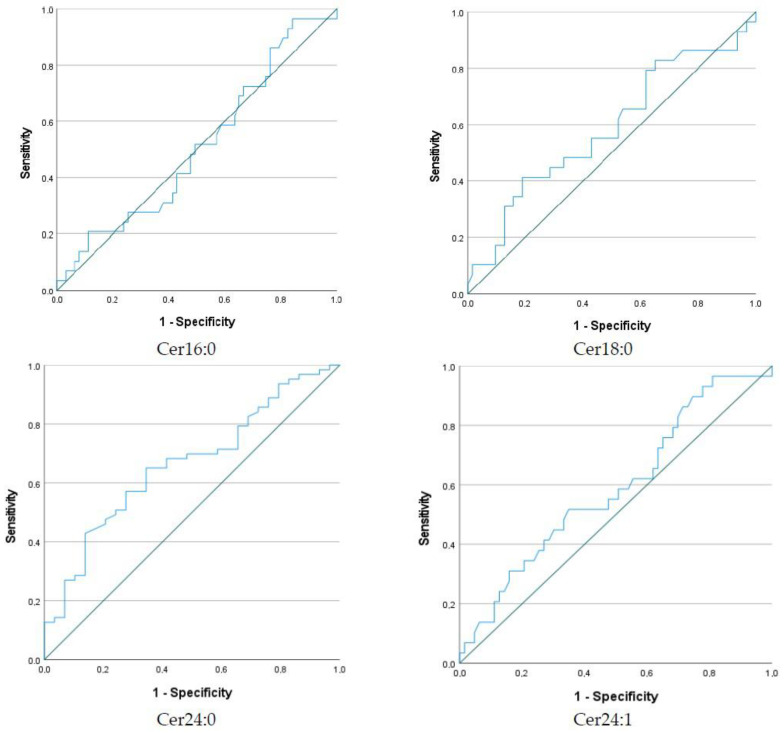
Receiver operating characteristic (ROC) curves for the four measured ceramides to predict GDM status.

Univariate statistical analysis demonstrated that the Cer24:0 concentration was significantly lower in the GDM group compared to the control group (*p* = 0.01). Figure 1 illustrates box plots for all ceramides in the two groups. In the ROC curve analysis, AUCs were calculated for Cer16:0 (AUC 0.5, 95% CI 0.383–0.636), Cer18:0 (AUC 0.6, 95% CI 0.455–0.716), Cer24:0 (AUC 0.7, 95% CI 0.547–0.778) and Cer24:1 (AUC 0.6, 95% CI 0.463–0.713) (Figure 2). A threshold Cer24:0 concentration of 4969.5 ng/mL had moderate sensitivity (69.8%) and specificity (51.7%) for the prediction of GDM status (GDM or control).

Furthermore, possible correlations between the serum concentration of ceramides with several parameters (maternal, delivery, neonatal, blood pressure, biochemistry) were investigated. No significant associations were found in the cohort of studied women (GDM and control groups) (Table 3) or the GDM (Supplementary Table S1) or control group (Supplementary Table S2) alone.

## 4. Discussion

This is the first study to quantify plasma ceramide concentrations in women with GDM and women with uncomplicated pregnancies at delivery. The study revealed that Cer24:0 concentrations were significantly decreased in women with GDM, while there were no differences in Cer16:0, Cer18:0, and Cer24:1 between GDM and control groups. These results are not the expected ones based on our initial hypothesis about the similar mechanisms of T2DM and GDM and elevated ceramides as biomarkers in patients with T2DM. On the other hand, our results partially agree with the results of three recent publications about the association of ceramides and GDM in early pregnancy [16,17]. More research is necessary to determine the mechanisms that affect circulating ceramides in pregnant women.

According to Liu et al. who included 486 women, in early pregnancy, high Cer18:0 and Cer18:1 and low Cer24:0 (≤3.60 nmol/mL) have been associated with higher GDM risk [odds ratio (OR) 1.69, 1.72 and 3.59, respectively] [16]. In another large, nested case-control study, including 1008 women, in which the lipidome of first-trimester pregnant women was studied, Cer24:0 and Cer24:1 were inversely associated with GDM [17]. Another recent study, including 135 women, which compared the ceramide concentrations in the first and the second trimester between pregnant women with normal glucose tolerance and women with GDM reported a significantly increased concentration of Cer18:1 in the second trimester in the GDM group [18]. These findings partially agree but are not enough to establish ceramides as clinical biomarkers in early pregnancy.

According to a point of view, GDM not only has similar risk factors but also similar molecular mechanisms with T2DM [19]. Maybe the role of ceramides in T2DM can enlighten the role of ceramides in GDM.

Several mechanisms have been proposed for the role of ceramides in diabetes mellitus (DM). The key to ceramides’ biological action may be their double biosynthesis: from hydrolysis of sphingomyelin by sphingomyelinases or de novo. There is a strong link between ceramide accumulation and insulin resistance. In rats, sphingolipids inhibit glucose transport into 3T3-L1 adipose fibroblasts. Ceramides reduce the insulin-induced tyrosine phosphorylation of insulin receptor substrate-1 (IRS-1). Lipotoxicity, defined as increased muscle, liver and plasma fat, plays a key role in the pathogenesis of T2DM. Ceramides, and other bioactive lipids, are related to lipotoxicity and inflammation [20]. Apoptosis and differentiation inducers, damaging agents and inflammatory cytokines increase ceramide production [7]. In addition, ceramides are the second major messengers of the inflammatory response induced by tumor necrosis factor-α (TNFα). Finally, increased Rac activation has been reported, and as a result, reduced glucose transferase-4 (GLUT-4) translocation to the plasma membrane in response to insulin [21].

Ceramide concentrations are elevated in the human adipose tissue of obese men and women compared to lean individuals [22]. A strong positive correlation was found between ceramide and TNFα concentrations in adipose tissue and a negative correlation between ceramide concentrations and adiponectin [23]. The Dallas Heart Study was the first large study (*n* = 1557) to reveal a positive correlation between shorter-chain saturated fatty acid ceramides (Cer18:0, Cer16:0, Cer20:0) with unfavorable adiposity, and lipid and insulin resistance; nevertheless, plasma ceramides were not independently associated with T2DM after adjustment for clinical factors [9]. A meta-analysis of 2337 participants from the Strong Heart Study and the Strong Heart Family Study showed higher plasma ceramide concentrations (Cer18:0, Cer20:0, Cer22:0) in patients who developed T2DM later. Possible mechanisms include ceramides inhibiting insulin intermediates such as insulin receptor substrate, protein kinase B (Akt) and GLUT-4, promoting β-cell apoptosis and dysfunction [24]. According to the same study, elevated plasma ceramides were correlated with a higher homeostatic model assessment of insulin resistance (HOMA-IR), a proxy of insulin resistance [25]. Based on these findings, ceramides are considered a promising biomarker for T2DM and a possible therapeutic target [26].

Recently, ceramide concentrations have been studied in several pregnancy complications, such as preterm labor, preeclampsia, hemolysis, elevated liver enzymes and low platelets (HELLP) syndrome, intrahepatic cholestasis, and even genetic disorders such as trisomy 21 [27]. Specifically, ceramide concentrations were higher in women with HELLP syndrome and a correlation was found with proteinuria [28]. The role of ceramides in preeclampsia is not yet clarified as there are conflicting results in the literature [11]. Maybe this is because, in the placental blood vessels of pregnancies complicated with preeclampsia, sphingolipid biosynthesis is shifted toward sphingomyelin rather than ceramides [29]. This molecular mechanism and the complex way of ceramide biosynthesis may explain the findings of the current studies. Furthermore, long-chain ceramides, such as Cer24, may have a different, benign, protective role in the vessels, contrary to Cer16 and Cer18 which contain C16 and C18 acyl chains [30]. Maybe the key to using ceramides as biomarkers is in creating ceramide scores, such as Cer18/Cer24 and a more individualized approach taking into consideration age, diet and BMI [31]. Further studies are needed to define the role of plasma ceramides in both uncomplicated pregnancies and pregnancies complicated by GDM.

Other studies provide possible mechanisms that can result in the elevation of ceramide production in preeclampsia [32]. Cer16:0 was found to increase in women with preterm labor [33]. Finally, plasma ceramides were found to increase in pregnant and lactating women compared to non-pregnant women; this finding supports the hypothesis that ceramides contribute to normal insulin resistance during pregnancy [34].

Even though a sample size estimation had been performed, we decided to include a larger number of participants in the study. Considering the small differences in the ceramide concentrations found between the two groups, the larger sample sizes proved to be essential. Although this is one of the first studies to suggest an association between ceramides and GDM, some limitations should be mentioned. The first is that ceramide concentrations were measured only once at delivery; no measurements during the first and second trimesters were available. Thus, the change in concentrations during pregnancy progression cannot be evaluated. Another limitation was the type of study: a cohort study would be more suitable than a case-control study. Finally, the potential molecular pathways by which ceramides are produced in GDM were not studied.

In conclusion, this study demonstrated a lower Cer24:0 plasma concentration during delivery in women with GDM compared to uncomplicated pregnancies. Our findings seem to partially agree with recent research about ceramides as possible biomarkers for GDM in early pregnancy. It has not yet been clarified whether ceramides are involved in metabolic disease pathways, or whether their abnormally increased biosynthesis leads to adverse effects. Further research is needed to establish a role for ceramides in everyday clinical practice.

## Figures and Tables

**Table 1:** Baseline parameters of study participants.

	GDM Group (*n* = 29)	Control Group (*n* = 63)	*p*-Value
Maternal age (years)	33.0 ± 4.5	30.0 ± 6.6	**0.029**
Gestational age (weeks)	37.9 ± 2.5	38.3 ± 1.1	0.288
Weight (kg) Pregestational BMI (kg/m^2^)	84.0 ± 16.0 26.7 ± 3.2	79.9 ± 13.2 23.9 ± 2.2	0.204 <**0.001**
Term BMI (kg/m^2^)	31.0 ± 4.1	29.6 ± 4.0	0.187
Gestational weight gain (kg) Neonatal birth weight (gr)	10.5 ± 5.8 3162 ± 761	15.3 ± 5.5 3190 ± 387	**<0.001** 0.815
Route of delivery			
Vaginal	12 (41.4%)	33 (52.4%)	0.327
Cesarian section	17 (58.6%)	30 (47.6%)	
Delivery indication			
Spontaneous delivery	17 (58.6%)	34 (54.0%)	0.774
Previous cesarian section	9 (31.0%)	19 (30.2%)	
Other obstetrical indication	3 (10.3%)	10 (15.9%)	
Medication			
None	0	63 (100%)	**<0.001**
Diet	17 (58.6%)	0	
Insulin	12 (41.4%)	0	
Smoking			
Smokers	6 (20.7%)	11 (17.5%)	0.711
Non-smokers	23 (79.3%)	52 (82.5%)	
ICU hospitalization of neonates			
Yes	2 (6.9%)	1 (1.6%)	0.183
No	27 (93.1%)	62 (98.4%)	

Results are given as mean ± standard deviation or as number (percentage). BMI: body mass index; *p*-values marked with bold indicate statistically significant differences between the groups GDM: gestational diabetes mellitus.

**Table 2:** Mean ceramide concentrations (ng/mL) ± standard deviation in GDM and control groups and *p*-values.

Ceramides (ng/mL)	GDM Group (*n* = 29)	Control Group (*n* = 63)	*p*-Value
Cer16:0	513 ± 226	489 ± 148	0.557
Cer18:0	224 ± 121	192 ± 84	0.149
Cer24:0	5032 ± 1487	6224 ± 2223	**0.010**
Cer24:1	3053 ± 1317	2673 ± 978	0.125

GDM: gestational diabetes mellitus. *p*-values marked with bold indicate statistically significant differences between the groups

**Table 3:** Correlation among ceramide concentrations and studied parameters for all studied women (GDM and control groups).

	Cer16:0	Cer18:0	Cer24:0	Cer24:1
Maternal parameters				
Age (years)	0.082	0.090	0.090	0.124
Weight (kg)	−0.025	0.005	−0.015	−0.095
Term ΒΜΙ (kg/m^2^)	0.097	0.079	0.124	0.077
Smoking	−0.061	0.034	0.025	0.059
Medications	0.029	0.139	−0.247	0.169
Delivery parameters				
Gestational age (weeks)	−0.008	−0.101	0.116	−0.069
Delivery mode	0.046	0.028	−0.086	0.130
Delivery indication	−0.018	0.055	−0.218	−0.152
Neonatal parameters				
Birthweight (g)	0.016	−0.065	0.089	−0.102
Apgar score at 1 min	−0.202	−0.259	−0.037	−0.014
Apgar score at 5 min	−0.261	−0.320	−0.009	0.010
Blood pressure				
SBP (mm Hg)	0.092	0.054	−0.008	0.104
DBP (mm Hg)	0.105	0.090	0.080	0.036
Biochemistry				
Creatinine (mg/dL)	0.066	0.043	−0.119	0.095
Urea (mg/dL)	−0.042	0.013	−0.199	0.011
SGOT (IU/L)	0.263	0.376	−0.062	0.045
SGPT (IU/L)	0.182	0.331	−0.111	−0.011
Uric acid (mg/dL)	0.174	0.151	−0.129	0.079
Platelets (10^3^/mL)	0.047	0.080	0.057	0.006

Data are given as Spearman correlation coefficient (ρ). BMI: body mass index; DBP: diastolic blood pressure; GDM: gestational diabetes mellitus; SBP: systolic blood pressure; SGPT: glutamate–pyruvate transaminase.

## Data Availability

The data presented in this study are available in the article.

## Supplementary Materials

The following supporting information can be downloaded at: https://www.mdpi.com/article/10.3390/metabo12111123/s1, Table S1: Correlation among ceramide concentrations and studied parameters for the GDM group; Table S2: Correlation among ceramide concentrations and studied parameters for the control group.
