# Supplementary material for: Plasma Ceramide Concentrations in Full-Term Pregnancies Complicated with Gestational Diabetes Mellitus: A Case-Control Study
Source: Metabolites. 2022 Nov 16;12(11):1123. doi: 10.3390/metabo12111123 (PMC9698071; doi:10.3390/metabo12111123)
Supplement: Supplementary file 1 [file metabolites-12-01123-s001.zip › metabolites-1984533-supplementary.pdf]

Table S1. Correlation among ceramide concentrations and studied parameters for the GDM group.

	**Cer16:0**	**Cer18:0**	**Cer24:0**	**Cer24:1**
Maternal parameters				
Age (years)	−0.066	0.097	0.038	0.147
Weight (kg)	−0.088	0.001	0.110	−0.124
Term BMI (kg/m^2)	0.130	0.180	0.540	0.498
Smoking	0.003	0.079	0.023	0.163
Medications	−0.100	−0.003	−0.005	0.081
Delivery parameters				
Gestational age (weeks)	−0.046	−0.116	−0.025	−0.099
Delivery mode	0.148	−0.003	0.107	0.207
Delivery indication	0.158	0.173	−0.052	−0.097
Neonatal parameters				
Birthweight (g)	−0.002	−0.066	−0.008	−0.150
Apgar score at 1 min	0.037	−0.061	−0.156	−0.004
Apgar score at 5 min	0.110	−0.077	0.153	0.067
Blood pressure				
SBP (mm Hg)	0.163	0.121	0.041	0.035
DBP (mm Hg)	0.204	0.265	0.157	0.298
Biochemistry				
Creatinine (mg/dL)	0.159	0.099	0.059	0.057
Urea (mg/dL)	−0.187	−0.115	−0.011	−0.009
SGOT (IU/L)	0.613	0.524	0.331	0.177
SGPT (IU/L)	0.679	0.651	0.394	0.224
Uric acid (mg/dL)	0.150	0.038	0.289	−0.050
Platelets (10^3/mL)	0.073	0.072	−0.154	0.064

Data are given as Spearman correlation coefficient (ϱ). BMI: body mass index; DBP: diastolic blood pressure; GDM: gestational diabetes mellitus; SBP: systolic blood pressure; SGPT: glutamate–pyruvate transaminase.

Table S2. Correlation among ceramide concentrations and studied parameters for the control group.

	**Cer16:0**	**Cer18:0**	**Cer24:0**	**Cer24:1**
Maternal parameters				
Age (years)	0.139	0.040	0.196	0.068
Weight (kg)	0.011	−0.026	−0.013	−0.118
Term BMI (kg/m^2)	0.073	0.015	0.088	−0.048
Smoking	−0.114	−0.007	0.042	−0.018
Medications	-	-	-	-
Delivery parameters				
Gestational age (weeks)	0.071	−0.040	0.180	0.016
Delivery mode	−0.032	0.023	−0.113	0.062
Delivery indication	−0.124	0.005	−0.301	−0.173
Neonatal parameters				
Birthweight (g)	0.046	−0.059	0.161	−0.042
Apgar score at 1 min	−0.345	−0.393	0.011	−0.037
Apgar score at 5 min	−0.448	−0.484	0.008	−0.034
Blood pressure				
SBP (mm Hg)	0.055	0.021	−0.024	0.149
DBP (mm Hg)	0.061	0.010	0.062	−0.085
Biochemistry				
Creatinine (mg/dL)	0.013	0.002	−0.148	0.105
Urea (mg/dL)	0.049	0.082	−0.249	0.010
SGOT (IU/L)	0.091	0.352	−0.203	0.007
SGPT (IU/L)	0.100	0.336	−0.194	−0.052
Uric acid (mg/dL)	0.202	0.214	−0.217	0.136
Platelets (10^3/mL)	0.017	0.073	0.172	−0.056

Data are given as Spearman correlation coefficient (ϱ). BMI: body mass index; DBP: diastolic blood pressure; SBP: systolic blood pressure; SGPT: glutamate–pyruvate transaminase.
